# Integrating Remote Monitoring Into Pregnancy Care: Perspectives of Pregnant Women and Healthcare Professionals

**DOI:** 10.1097/CIN.0000000000001255

**Published:** 2025-02-05

**Authors:** Likitalo Susanna, Pakarinen Anni, Axelin Anna

**Affiliations:** Department of Nursing Science, University of Turku, Finland

**Keywords:** Maternal health services, Obstetrics, Pregnancy, Remote monitoring, Telemedicine

## Abstract

Remote monitoring has been proposed to provide new opportunities to monitor pregnancy in the home environment and reduce the number of follow-up visits to the maternity clinic. Still, the integration of remote monitoring into the pregnancy care process has not been achieved. This descriptive qualitative study aimed to explore pregnant women’s and healthcare professionals’ perceptions of integrating remote monitoring into pregnancy monitoring process. A convenience sample of 10 pregnant women and 11 healthcare professionals participated in the focus group interviews. The data were analyzed with reflexive thematic analysis. The results comprised a four-step pregnancy monitoring process organizing the issues to consider when integrating remote monitoring into these steps. According to pregnant women and healthcare professionals, remote pregnancy monitoring should allow a holistic assessment to ensure the well-being of the pregnant woman and the fetus. Clear criteria for monitoring should guide the adaptation of monitoring to the identified monitoring needs. Ideally, remote monitoring could enable more personalized maternity care, supporting the monitoring-related decision-making of both pregnant women and healthcare professionals and facilitating the early detection of pregnancy complications. However, integration of remote monitoring would require significant restructuring of current pregnancy care processes.

The digitalization of healthcare is increasing.^1^ Reviewing the healthcare system and individual care paths is needed to identify the most appropriate application points for technology.^2^ Technology and digital tools are already an integral part of people’s daily lives, and the World Health Organization encourages focusing on utilizing these technological solutions to promote healthy living.^3^ As health promotion and ensuring the health of both the pregnant woman and the fetus play an important role in maternal care,^4^ the potential of digital services is also recognized as part of pregnancy care.^5^ In particular, the growing need to develop high-quality healthcare services with diminishing resources has increased the interest in utilizing technology as part of healthcare.^6,7^

One way to utilize technology is to improve the monitoring possibilities by remote monitoring solutions. Remote monitoring refers to using digital devices outside the healthcare setting. Remote measurements are transferred to the healthcare system to be able to use them as part of care.^8^ Some solutions have already been developed to use remote monitoring to provide pregnancy care in the home environment, such as supplementing clinical appointments with virtual meetings and remote blood pressure measurements.^9^ However, implementing and integrating digital services into clinical workflows is one of the significant challenges associated with using technology.^10,11^ Even solutions that are found to be effective may not survive if the implementation and sustainable use are not considered already during the development process.^12^ Especially in pregnancy care, for example, the need for physical examination during pregnancy and reduced empathy during remote appointments are considered as barriers to technology implementation.^13^

In addition, the characteristics of the developed technology may affect the opportunities to implement the solution. For example, solutions that focus only on individual pregnancy complications may not be able to address the broader need for pregnancy monitoring.^14^ To ensure that the developed technological solution meets its intended use as well as possible, it is important to become familiar with the context in which the device will be used.^15^ One way to facilitate the implementation and integration of the technologies is to involve the relevant stakeholders in development and research processes.^10,16^ Stakeholders would provide a comprehensive view of the targeted health condition, end-users, their needs, and the context, such as the healthcare system. Involving end-users helps to identify components and actors of the system and the relationships between them that might otherwise be overlooked.^2^ Familiarizing with stakeholders’ experiences and views during the development process could help to identify factors affecting the use of the devices and allow them to be considered during the development phase.

The aim of this study was to explore the perceptions of pregnant women and healthcare professionals on integrating remote monitoring into the pregnancy monitoring process.

## METHODS

### Design and Setting

This descriptive qualitative focus group study^17,18^ was conducted in healthcare units, such as maternity clinics and hospital wards, participating in pregnancy care in the area of the Wellbeing Services County of Southwest Finland (Varha). In the Varha area, there are 68 maternity clinics in 27 municipalities and one maternity hospital. Approximately 4000 babies are born in this hospital every year.

In Finland, pregnancy care and follow-up visits are carried out in close cooperation between the maternity clinics and the hospital. In the maternity clinic, the follow-up visits are provided by public health nurses or midwives and physicians. The national guidelines^19^ provide the basis for pregnancy monitoring and follow-up practices guaranteeing equal minimum services, regardless of where the pregnant woman lives. For first-time mothers, a normal pregnancy follow-up consists of a minimum of nine visits in the maternity clinic. For pregnant women with previous childbirths, the minimum number is eight visits. Two of the visits include a medical examination by a physician, whereas the public health nurse or midwife performs other visits. During each follow-up visit, the health of the pregnant woman, fetus, and the whole family is assessed comprehensively. In addition to the health discussion, the follow-up visits consist of different measurements, such as measuring blood pressure and assessing fetal heart rate and uterine growth. Pregnancy monitoring is usually implemented during physical follow-up visits in the healthcare environment. Still, in the case of increased risk for pregnancy complications, there are possibilities to monitor, for example, blood pressure and blood glucose levels at home, with blood pressure or blood glucose monitors. In addition to the routine follow-up visits, pregnancy follow-up is supplemented by additional visits to a maternity clinic or, in the case of possible deviations, to a hospital.

### Participants

Convenience sampling was used to obtain a credible understanding of the research topic. To obtain diverse views on remote pregnancy monitoring, the study involved pregnant women and healthcare professionals from different occupational groups and healthcare units. The final sample size was based on the data saturation evaluated during the data analysis.^18^

The inclusion criteria for pregnant women were (1) being at least 29 weeks pregnant and (2) being able to participate in a remote interview implemented in Finnish. The inclusion criteria for healthcare professionals were (1) working in pregnancy care and (2) being able to participate in a remote interview implemented in Finnish.

### Data Collection

Data were collected with focus group interviews using a semistructured interview guide to encourage participants to express diverse views stimulated by other participants.^18^ The interview guide (Appendix 1, http://links.lww.com/CIN/A410) consisted of three themes: (1) the current practices and pregnancy care path; (2) possibilities of improving follow-up practices with remote monitoring; and (3) requirements and wishes for remote monitoring. In addition, pregnant women were asked about the duration of the pregnancy and the number of children. Healthcare professionals were asked about their occupation, workplace, and working experience in that unit. The data were collected between June and September 2023.

### Procedure

In recruiting pregnant women, healthcare professionals of the pregnancy follow-up ward first briefly informed the eligible pregnant women about the study, after which the researcher gave additional information to those who expressed interest in the study. Healthcare professionals were recruited via work email and with the help of head nurses of different healthcare units. Pregnant women gave the written informed consent after receiving the additional information about the study, and healthcare professionals gave the informed consent electronically via link in the recruitment message. After giving informed consent, the researcher agreed with the participants on an appropriate interview time.

Focus group interviews were organized separately for pregnant women and healthcare professionals, and one to four interviewees participated in each focus group interview simultaneously. The focus group interviews were conducted remotely with a Zoom connection, each lasting approximately 60 minutes. The focus group interviews were audio-recorded and transcribed verbatim. The same researcher performed all the focus group interviews, and another research group member operated as a facilitator. Preliminary data analysis and evaluation of the data saturation were performed simultaneously with the focus group interviews. In the middle of the data collection, the discussed themes began to repeat, and in the case of the last focus group interviews, no new subthemes were identified, and data saturation was achieved.

### Data Analysis

The transcribed data were analyzed using the reflexive thematic analysis.^20^ First, the transcribed data were read several times to understand the process of pregnancy monitoring and pregnancy care where remote monitoring would be integrated. Based on the data, the monitoring process was divided into four phases, which were named following a previously identified nursing process^21^: assessment and identification, planning, implementation, and evaluation. This structure provided the lens for further analysis and interpretation. After that, the analysis continued by generating the initial codes from the data to select interesting and meaningful features (sentences or combinations of words) for the analysis. The produced codes were sorted into the initial subthemes. Originally, the focus group interview data of the pregnant women and the healthcare professionals were analyzed separately. However, when similar subthemes were identified from both data, they were combined where applicable before the final refinement of the analysis. Finally, the codes and the subthemes were refined to form a thematic map describing the data within the four main phases of the pregnancy monitoring process. An individual researcher was conducting the analysis because multiple coders are not required in reflexive thematic analysis to ensure the quality of the analysis.^22^ However, all decisions related to forming the thematic map were discussed with other authors to enhance the trustworthiness of the analysis. Because the researcher conducting the analysis had a working career in maternity care, she continuously reflected her own influence on the analytic process. This aimed to ensure that her own experiences did not interfere with the interpreting and capturing of the shared meanings of the participants.^20^ In the reporting phase, the quotes from the participants were translated from Finnish into English. To maintain the original meaning of the quotes, the translations were compared with the original quotes and refined where needed.

### Ethical Considerations

Ethical approval was obtained for the study from the Ethics Committee for Human Sciences at the University of Turku (25/2023) in May 2023. Research permission was obtained from the Wellbeing Services County of Southwest Finland in June 2023.

## RESULTS

Altogether, 11 focus group interviews were conducted, and 10 pregnant women and 11 healthcare professionals participated in the focus group interviews. The pregnant women were primiparas and multiparas. The pregnancy weeks of the participants varied between 29 + 2 and 37 + 4. One of the healthcare professionals was a physician, eight were midwives, and two were public health nurses working in the maternity clinic. The working experience of the participants varied from 8 months to 23 years.

**FIGURE 1:**
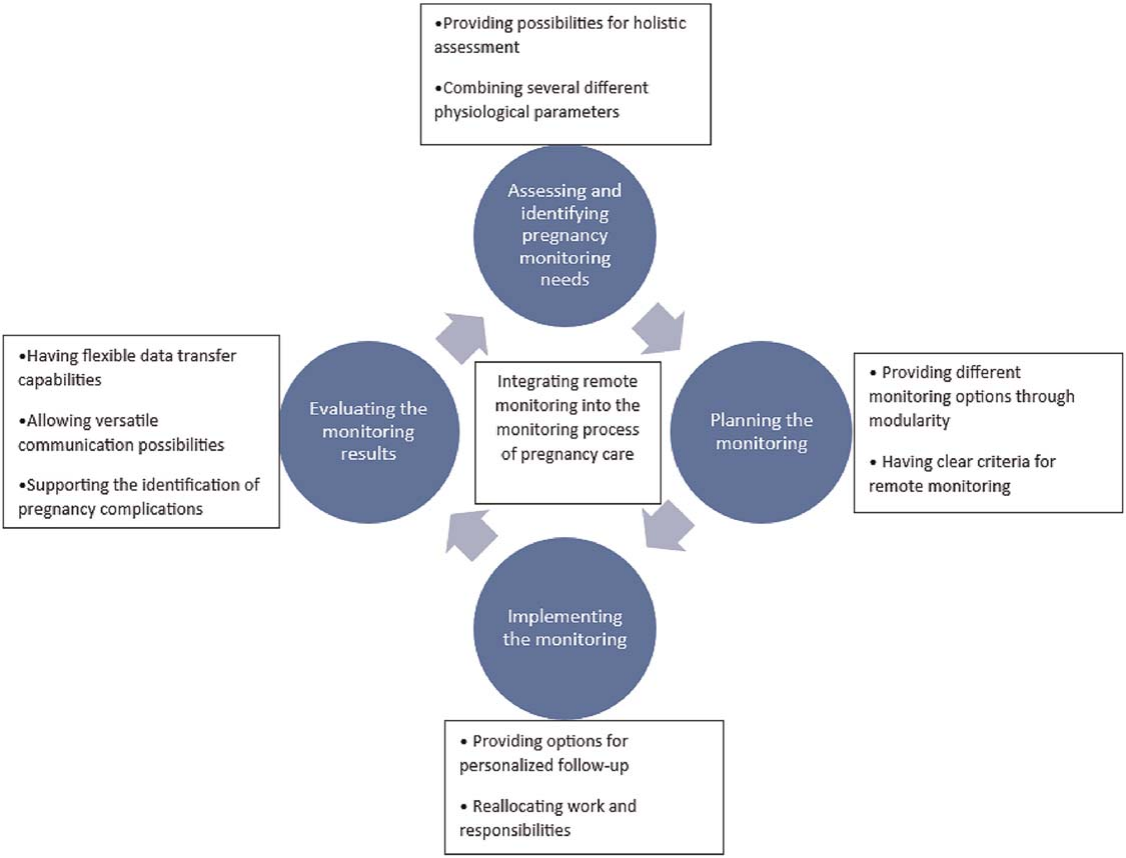
Pregnant women’s and healthcare professionals’ perceptions on integrating remote monitoring into the monitoring process of pregnancy care.

The pregnancy monitoring process was described as having four phases repeated continuously throughout the pregnancy: assessing and identifying the pregnancy monitoring needs, planning the monitoring, implementing the monitoring, and evaluating the monitoring results. Altogether, nine subthemes were identified to supplement these four main themes and describe the participants’ perceptions of integrating remote monitoring into the monitoring process of pregnancy care (Figure 1).

### Assessing and Identifying Pregnancy Monitoring Needs

The pregnancy monitoring process started with assessing and identifying the existing monitoring needs by gathering information about the overall situation of the pregnant woman, fetus, and the whole family. To reinforce the consideration of different monitoring parameters, the participants suggested that remote monitoring should provide possibilities for holistic assessment. It was not enough to monitor physiological parameters, but must also look at the pregnant woman’s overall well-being and mental health. These could be conducted, for example, by measuring physical activity or assessing the symptoms of antenatal depression. In addition, healthcare professionals stressed the importance of social parameters in assessing the family’s overall situation to evaluate the living environment of the unborn child.

Pregnancy monitoring is monitoring of health. Both physiological health and to some extent also the mental health. One aspect is also to find out the social things. (Healthcare professional)

However, the physiological parameters were still considered a key issue when assessing the potential onset of pregnancy complications. To get an overview of the health situation of the pregnant woman and the fetus, the participants suggested that remote monitoring should combine several different physiological parameters. The participants described that if the monitoring focused only on individual parameters, there would not be enough information to identify more complex pregnancy conditions. For example, in preeclampsia, symptoms may include high blood pressure, proteinuria, edema, severe headaches, or other subjective symptoms. Focusing only on blood pressure, for example, could lead to other symptoms of preeclampsia being overlooked. According to the participants, a remote monitoring system was required to evaluate the measurements of the pregnant woman and the fetus together, as changes in the pregnant woman’s condition might affect the well-being of the fetus. In addition, the participants stressed the importance of reliable measurement results in using remote monitoring as part of pregnancy care.

Pregnancy monitoring needs to be more comprehensive. Only one parameter is not enough to make the assessment. (Healthcare professional)

### Planning the Monitoring

The participants described that the monitoring needs usually varied throughout the pregnancy and from one pregnant woman to another. Planning pregnancy monitoring to meet these varying needs requires high adaptability of the remote monitoring system. Remote monitoring was described to meet these adaptation needs by providing different monitoring options through modularity of the system. In addition to the normal changes in the monitoring needs according to the weeks of pregnancy, the participants also described the need for rapid modifications due to sudden and unpredictable events such as premature rupture of the membranes or pregnancy complications. All these changes were described as impacting the required parameters to be monitored. As the situation changes, the remote monitoring system should be equally easy to scale down when increased monitoring is no longer required.

Last week there was a little bit of surprise with bleeding which intensified the monitoring. But now the situation is stable again, and we continue according to the normal follow-up plan. (Pregnant woman)

According to the participants, planning an appropriate remote monitoring setup to support pregnancy follow-up required clear criteria. The criteria were described to include the limit values for parameters indicating possible pregnancy complications and also the individual preferences of pregnant women related to how the monitoring should be implemented. Healthcare professionals expressed concern that remote monitoring might lead to excessive oversight and unnecessary interference in the course of pregnancy. To prevent this, they proposed establishing clear criteria to ensure that monitoring is always justified and that actions during remote monitoring are based on precise measurements. The participants also pointed out that some pregnant women might find remote monitoring and increased data stressful. Considering the individual preferences of the pregnant woman in question, such as willingness to remote monitoring, would also help to choose the most appropriate follow-up practices for each pregnant woman.

When considering remote monitoring, it would need clear criteria to decide for whom you give that kind of a monitoring device since it should be beneficial for both us and for pregnant women. (Healthcare professional)

If there is an identified need for monitoring, then remote monitoring could provide the sense of security. (Pregnant woman)

### Implementing the Monitoring

When implementing the planned pregnancy monitoring, the participants considered remote monitoring to provide options for personalized follow-up. The participants suggested that remote monitoring could provide more versatile follow-up options for traditional visits. Some pregnant women, and especially the nonpregnant partners, sometimes had difficulties organizing access to on-site follow-up visits, for example, due to work commitments or distance from the healthcare organization. Remote appointments, independent of time and place, would provide more opportunities for participation. It was also considered that pregnant women with previous childbirths or at low risk of pregnancy complications might prefer remote monitoring options to traditional visits. The healthcare professionals also suggested that having some remote monitoring data between the follow-up visits could help them react to possible deviations promptly, reducing the need for frequent follow-up visits. They also considered that having monitoring data before the follow-up visit could help them to tailor the content of the next visit to better meet the individual needs of the pregnant woman. Remote measurements could also allow more time for discussion between the pregnant woman and the healthcare professional during the visit. However, some of the participants were concerned that increased remote monitoring could threaten the physical encounter and therefore the emotional support that healthcare professionals provide to pregnant women. This confirmed participants’ view that remote monitoring should be used to provide additional or parallel follow-up options, not to replace physical visits completely.

Also, there should be face-to-face service available, when needed. These two options should go side-by-side. (Healthcare professional)

If there was a very basic monitoring and reliable device and connections, I would have preferred to skip some of the travels to the hospital. So I consider it also as a practical thing, when you don’t have to take off half a day to visit the hospital. (Pregnant woman)

In implementing pregnancy care including remote monitoring, the participants highlighted the need to reallocate work and responsibilities between different actors, such as different healthcare professionals and pregnant women. They suggested that remote monitoring could help to reduce the overlap between maternity clinic and hospital follow-up visits and refine the division of work between these care providers. They felt that remote monitoring could, for example, reduce the need for hospital referrals if the public health nurse in the maternity clinic could send the data to be evaluated by the healthcare professionals in the hospital.

There are referrals to the hospital where a pregnant woman has visited maternity clinic 2 hours earlier, and the fetus has had a high heart rate. It’s possible that the fetus has been moving a lot at that moment. So, I think that this kind of referrals could be reduced if they could take some heart rate pattern in the maternity clinic. (Healthcare professional)

In terms of responsibilities, the participants also described the role of the pregnant woman. The participants described that pregnant women would need clear instructions on using the remote monitoring system and reacting to the deviations in the monitored parameters. This would require a clear protocol of who receives and interprets the measurements and when. It was suggested that healthcare professionals should be responsible for interpreting the measurements to ensure the safety of remote monitoring. At the same time, pregnant women should be more responsible for using remote monitoring according to the instructions provided.

It would be nice to, for example, listen the fetal heartbeat at home, but interpreting it would require special knowledge. So it would be good if some professionals could also see the heart rate pattern. (Pregnant woman)

### Evaluating the Monitoring Results

To be able to evaluate the monitoring results, remote monitoring requires flexible data transfer capabilities. The participants described that due to the diverse cooperation between the pregnant woman and different healthcare professionals in the maternity clinic and the maternity hospital, there should be a possibility to share the data with all relevant actors involved in pregnancy care. The results should be available also to the pregnant woman herself. However, instead of expecting the pregnant woman to transfer the pregnancy-related information between different healthcare units, there should be a possibility to share the information directly from the remote monitoring system. The pregnant women also suggested that they should be able to share the information with their nonpregnant partner, if they so wished, to support their involvement in the pregnancy.

After all, we have quite a lot of cooperation which requires transferring the data. During the pregnancy, pregnant woman might visit the laboratory, maternity clinic and hospital all in different locations, so it’s pretty essential that the needed information reaches the right professionals. (Healthcare professional)

Or maybe there could be different levels for sharing data. If the pregnant woman wants, there could be possibilities to share the data with the spouse. (Pregnant woman)

In addition to effective data transfer, the participants identified a need for versatile communication possibilities between the different actors involved to support the evaluation of the monitoring results. There should be easy communication between the healthcare professionals to facilitate the consultation between different specialists and reciprocal communication between the pregnant woman and the healthcare professionals. This would allow pregnant women to ask for help or support and healthcare professionals to receive further information from the pregnant woman. In addition, healthcare professionals considered the potential benefits of enhanced communication in terms of increasing the fluency of pregnancy care. However, pregnant women and healthcare professionals stressed the importance of enabling communication between follow-up visits and facilitating access to healthcare. The participants suggested that one way of doing this could be through a real-time communication channel.

A channel providing easy connection to the public health nurse in maternity clinic. (Healthcare professional)

In terms of evaluating the monitoring results, the participants described the identification of pregnancy complications as one of the key objectives of the evaluation, requiring decision-making from both pregnant women and healthcare professionals. The pregnant women experienced the subjective assessment of, for example, fetal movements between the follow-up visits as difficult and stressful. They suggested that remote monitoring could be a tool to support this assessment. The system could provide a more objective assessment of the situation alongside their subjective views, increasing the pregnant woman’s sense of security. Also, healthcare professionals recognized the potential of remote monitoring in identifying deviations and monitoring needs. They considered that a remote monitoring system could help them, for example, to interpret the fetal heart rate patterns that might otherwise require consultation with another healthcare professional.

It could provide information about which results are good and which are not and when to contact someone. Also provide clarifications for pregnant woman so that the assessment of the condition wouldn’t be so much on her own responsibility. (Pregnant woman)

The device would analyze with you, giving you some certain criteria. So, you won’t be all alone… you can get like assistant to support your decision-making. (Healthcare professional)

## DISCUSSION

The aim of this study was to explore pregnant women’s and healthcare professionals’ perceptions of integrating remote monitoring into pregnancy monitoring process. Our findings describe issues to be considered when integrating remote monitoring into the different phases of the process. According to the participants, monitoring should allow for a holistic assessment to ensure the well-being of the pregnant woman and the fetus, even in the case of complex conditions. A precise set of criteria should guide the adaptation of monitoring to needs. Ideally, remote monitoring could enable more personalized maternity care. However, this would require significant restructuring of current care processes. According to the participants, effective remote monitoring has the potential to support shared decision-making and early detection of pregnancy complications.

To facilitate the assessment and identification of pregnancy monitoring needs, the remote monitoring system should combine several parameters to meet the holistic and diverse pregnancy monitoring needs. Even though, for example, perinatal mental health is acknowledged as an important perspective in pregnancy care,^23^ to the best of our knowledge, there are no solutions described in the previous literature where a holistic remote monitoring system combines various monitoring parameters. Instead, most solutions present the monitoring of individual physiological parameters, for example, blood pressure^9,24^ or blood glucose levels,^25^ to provide additional information for healthcare professionals. These individual parameters alone cannot meet holistic monitoring needs, considered one of the most central objectives of pregnancy care.^26^ In addition to pregnant women’s holistic care, pregnancy-related assessments should also consider the well-being of the fetus due to possible changes in the health condition of the fetus caused by pregnancy and pregnancy complications.^14,27^ Using remote monitoring to combine all the parameters needed and assess them with relevant algorithms would facilitate the effective identification of varying monitoring needs during pregnancy.

When planning the implementation of pregnancy monitoring, integrating remote monitoring into the pregnancy care path could provide more flexible and timely monitoring possibilities than traditional monitoring practices. Early detection of the symptoms is known to be important for many pregnancy complications, and remote pregnancy monitoring is suggested to support the early identification.^28,29^ By modularity and providing versatile monitoring possibilities, remote monitoring could reduce the need for routine additional visits and increase the monitoring even between the follow-up visits. The results of this study suggest that remote services should be designed into the system to run in parallel with conventional services without necessarily replacing the current follow-up visits, which is consistent with previous literature.^16,30^ However, these changes in the pregnancy care system would require carefully planned implementation^29^ and willingness to reconstruct the pregnancy follow-up system.

Although integrating remote pregnancy monitoring was identified to have many benefits for pregnant women, healthcare professionals, and for the provision of pregnancy care, it is also important to recognize the challenges associated with increased remote monitoring. One of the main concerns was the increased stress and worries among pregnant women due to the large amount of data, which is also identified in previous literature.^31,32^ The participants of this study also highlighted the risk related to too excessive monitoring and unnecessary interference during pregnancy. These challenges are also identified in previous literature. Increased fetal monitoring has been described to cause severe problems during childbirth, such as ending up in cesarean delivery due to false alarms indicating fetal distress.^33^ These aspects should be acknowledged also during pregnancy monitoring. Based on the results of this study, one way to facilitate this concern would be to define clear criteria for using remote monitoring and interpreting the measurements, which would ensure that the monitoring would always be based on an identified need. However, it is worth noting that pregnancy itself has been described as a stressful phase of life,^34^ so it would be important to consider the potential of remote monitoring in alleviating pregnancy-related stress as a whole.

Integrating remote monitoring in the implementation phase of the pregnancy monitoring process was suggested to provide major benefits in increasing the individuality and personalization of care. This finding is congruent with previous literature.^29,35^ The structure of the current pregnancy care varies between different countries but is still primarily based on predetermined physical follow-up visits intensifying toward the end of the pregnancy. This kind of structure leaves little room for individual preferences or wishes. As in this study, previous literature has also identified differences between pregnant women’s preferences regarding how often they want to visit the maternity clinic and how important they consider the encounter with a healthcare professional to be.^35^ Although some pregnant women might find arranging the follow-up visits difficult, for example, due to work demands or childcare arrangements,^36–38^ some were more concerned about the reduced physical encounter with healthcare professionals due to remote monitoring, which is also congruent with previous literature.^36^ Contrary to previous literature, the results of this study suggest that taking routine measurements already before the physical follow-up visit using remote monitoring could even save time for discussion during the follow-up visits, facilitating the encounter with a healthcare professional. Also, in contrast to previous literature, the results of this study identified remote monitoring as one possibility to increase the participation of the nonpregnant partner. Although the role of the nonpregnant partner is often overlooked, involving them in pregnancy care is known to be beneficial for the whole family,^39,40^ highlighting the need for considering the role of nonpregnant partners also during the development of remote pregnancy monitoring.

Although remote monitoring was suggested to facilitate the identification of pregnancy complications in the evaluation phase of the pregnancy monitoring process, the increasing amount of data has also raised concerns related to the increased workload of healthcare professionals.^32,41^ Based on the previous literature, at least part of the interpretation of the extensive amount of monitoring data could be facilitated by artificial intelligence.^28,32^ This possibility was also suggested by the participants of this study. However, even if the workload of the healthcare professionals could be facilitated, the use of remote pregnancy monitoring would still increase the role and responsibility of the pregnant woman.^26,38^ Remote monitoring in a home environment would require active participation from the pregnant woman, requiring sufficient support for her during the remote monitoring.^42^ From the pregnant women’s point of view, pregnancy-related self-monitoring was considered challenging, but monitoring possibilities between the follow-up visits could make them feel safer and more relieved, which is congruent with previous literature.^35^ In general, pregnant women desired more support between the follow-up visits to maternity care. As described in previous literature, remote monitoring with improved monitoring and communication was considered a potential way to facilitate that support.^32,43^ Increasing the pregnant woman’s responsibility in pregnancy care could also increase the feeling of involvement in care.^44^ Active participation in their own care would support patient engagement, which has been recognized to improve the quality of care.^45^ As pregnancy is considered a condition that should not be treated as a disease, it is somewhat natural and justified to give more responsibility to the pregnant woman herself. However, this kind of change in the division of responsibilities requires willingness from the healthcare professionals to pass some of the responsibilities from themselves to the pregnant woman, which they may not always be ready for.^35^

### Trustworthiness

In relation to the trustworthiness of the study and to obtain rich interview data, the researcher asked clarifying questions during the focus group interviews to engage the participants, facilitating the credibility of the study.^46^ Even though the researcher asked the participants to confirm their interpretations during the focus group interviews, asking them for feedback about the interpretations again after the analysis would have supported the trustworthiness of the study. However, involving both pregnant women and healthcare professionals in the focus group interviews increased the data triangulation. The initial goal was to include three to four participants in each focus group interview, but especially among pregnant women, the challenges with scheduling and unpredictable events during the late pregnancy caused last-minute cancellations of interviews. For that reason, the content of some focus group interviews might not be as diverse and rich as possible because the benefit of focus group interviews was not achieved. Also, including pregnant women and healthcare professionals in the same focus group interview would have elaborated the discussion. However, the end-user groups were kept separate to avoid possible tensions due to the power status. The representatives of different end-user groups can also be seen as a limitation. Although the representativeness of pregnant women was fairly good, most participating healthcare professionals were midwives. This may have made the hospital’s perspective more prominent in the results than the maternity clinic’s. In addition, only one physician participated in the focus group interviews, which makes it impossible to draw credible conclusions about the perceptions, especially of the physicians.

Although the description of the participants is limited in supporting transferability,^46^ the description of the study process and decisions related to data analysis are well documented, supporting the confirmability of the study.^46^ In reporting the study results, the Standards for Reporting Qualitative Research^47^ checklist was utilized to ensure that all relevant aspects were presented. When utilizing the results of this study, limited transferability due to varying healthcare systems between different countries needs to be considered. Although the objectives of pregnancy monitoring are similar, the relevancy of the aspects of the provided results needs to be assessed in relation to the healthcare system in question.

## CONCLUSION

Pregnant women and healthcare professionals identified remote pregnancy monitoring as potentially improving current pregnancy care and enabling new ways of implementing pregnancy monitoring. Remote monitoring could, for example, increase the personalization of care, support both professionals and pregnant women in monitoring-related decision-making, and contribute to the early identification of pregnancy complications. However, the successful integration of remote monitoring requires notable changes in pregnancy care processes in relation to the allocation of work and responsibilities between different parties in pregnancy care. In future research, various representatives from different levels of healthcare system providing pregnancy care should be involved to explore and clarify the required changes in pregnancy care process.

## Supplementary Material
