# Supplementary material for: Integrating Remote Monitoring Into Pregnancy Care: Perspectives of Pregnant Women and Healthcare Professionals
Source: Comput Inform Nurs. 2025 Feb 5;43(12):e01255. doi: 10.1097/CIN.0000000000001255 (PMC12704663; doi:10.1097/CIN.0000000000001255)
Supplement: Supplementary file 1 [file nxn-43-e01255-s001.docx]

## Semi-structured interview guide used in the focus group interviews

| Introduction |
| --- |
| Background of the pregnant women (e.g., phase/duration of the pregnancy, number of pregnancies) and healthcare professionals (e.g., occupation, working unit, duration of the career) |
| Theme 1. Understanding **the current practices and pregnancy care path** |
| During the interview, we will focus especially on monitoring during pregnancy, but we are also interested in monitoring the early stages of childbirth, when the pregnant woman may not yet be registered in the hospital/maternity ward.  The discussed topics:   - Essential objectives of pregnancy follow-up/monitoring - Progression of monitoring during pregnancy - Persons/professionals involved in the pregnancy care |
| Theme 2. Understanding the **possibilities of improving follow-up practices with remote monitoring** |
| The discussed topics:   - Perceptions related to using remote monitoring during pregnancy - Possibilities to improve current follow-up practices with remote monitoring - Possible effects on conducting monitoring at home or staying at home when monitoring is needed |
| Theme 3. Understanding the **requirements and wishes for remote monitoring** |
| With the remote monitoring device/system, we mean in this interview any device used by a pregnant woman outside healthcare and from which a health care professional receives some information about the results of the monitoring. So, at this point we have not defined the characteristics of the device.  The discussed topics:   - Possibilities and challenges related to moving care from hospital/healthcare setting to home - Requirements for using remote monitoring - Brainstorming the type and characteristics of potential device for remote pregnancy monitoring |
